# Supplementary material for: Association Between Melatonin Use and Cataract Risk: A Target Trial Emulation Retrospective Cohort Study
Source: Antioxidants (Basel). 2025 Aug 20;14(8):1016. doi: 10.3390/antiox14081016 (PMC12383021; doi:10.3390/antiox14081016)

Supplementary

Table S1. Key Components of Target Trial Protocol vs. Emulation Protocol

Protocol Components	Target Trial Protocol	Emulation Protocol
Aim	To compare the risk of cataracts between melatonin use and hypnotic benzodiazepines (BZD).	Same as the target trial.
Eligibility criteria	Adults aged ≥40 years with a diagnosis of sleep disorder and no prior history of lens diseases.	Adults aged ≥40 years with sleep disorder, ≥2 healthcare visits, and no prior history of lens diseases prior to index date, identified using the TriNetX database.
Treatment strategies	Initiate a melatonin or hypnotic BZD and continue to use through the follow-up period	Initiation of either melatonin or hypnotic BZD with ≥2 prescriptions at least 3 months apart
Assignment procedures	Randomized assignment to melatonin or hypnotic BZD.	By leveraging TriNetx's built-in capability for simulating randomization through propensity-based matching, this method enables the creation of representative target cohorts for randomized clinical trials.
Follow-up period	Follow-up begins at the time of treatment assignment and continues until study results become available, the participant dies, is lost to follow-up, or the end of the study period (up to 5 years).	Follow-up begins one year after treatment assignment and continues until the earliest of the following events: the availability of study results, death, loss to follow-up, or the end of the study period (up to five years). Additionally, we applied various lag times (e.g., no lag, one year, or two years) between the index date and the occurrence of cataract outcomes.
Outcome of interest	Primary outcome:<br>Incidence of age-related cataract and other cataract types<br>Secondary outcome:<br>Secondary outcomes included traumatic cataract, complicated cataract, drug-induced cataract, secondary cataract, other specified cataract, and unspecified cataract.	Same as the target trial.
Causal contrast of interest	Intention-to-Treat Analysis, ITT:	Intention-to-Treat Analysis, ITT:

	Treatment Definition: Patients who were assigned to either melatonin or hypnotic BZD on Day 0.<br>Effect Assessment: The analysis evaluates outcomes based on the baseline assigned treatment group, regardless of subsequent treatment adherence.	Treatment Definition: Patients with a prescription record for melatonin or hypnotic BZD on Day 0.<br>Effect Assessment: Outcomes are analyzed based on the baseline-assigned treatment group, regardless of subsequent treatment continuation.
Statistical analysis	Cox proportional hazards model	Same as the target trial.

Table S2. Relevant codes used for the cohort

**Inclusion criteria**	**Code system**	**Relevant codes**
Melatonin	RXNORM	6711
Hypnotic BZD	ATC	N05CD
Sleep disorders	ICD-10	G47
Ophthalmology Services and Procedures	CPT	1012793
**Exclusion criteria**	**Code system**	**Relevant codes**
Disorders of lens	ICD-10	H25-H28
**Variable**	**Code system**	**Relevant codes**
Diseases of the circulatory system	ICD-10	I00-I99
Hypertensive diseases	ICD-10	I10-I1A
Insomnia	ICD-10	G47.0
Anxiety, dissociative, stress-related, somatoform and other nonpsychotic mental disorders	ICD-10	F40-F48
Diabetes mellitus	ICD-10	E08-E13
Depressive episode	ICD-10	F32
Neoplasms	ICD-10	C00-D49
Ischemic heart diseases	ICD-10	I20-I25
Nicotine dependence	ICD-10	F17
Cerebrovascular diseases	ICD-10	I60-I69
Diseases of arteries, arterioles and capillaries	ICD-10	I70-I79
Alcohol related disorders	ICD-10	F10
Epilepsy and recurrent seizures	ICD-10	G40
Tobacco use	ICD-10	Z72.0
Parkinson's disease	ICD-10	G20
Type 2 diabetes mellitus with ophthalmic complications	ICD-10	E11.3
Alzheimer's disease	ICD-10	G30
Other hypnotics and sedatives	ATC	N05CM
Benzodiazepine related drugs	ATC	N05CF
**Outcome**	**Code system**	**Relevant codes**
Age-related cataract	ICD-10	H25
Other cataract	ICD-10	H26
Traumatic cataract	ICD-10	H26.1
Complicated cataract	ICD-10	H26.2
Drug induced cataract	ICD-10	H26.3
Secondary cataract	ICD-10	H26.4

Other specified cataract	ICD-10	H26.8
Unspecified cataract	ICD-10	H26.9
Urinary tract infection	ICD-10	N39.0

Table S3. The lag time analysis for the risk of cataract after index date among study cohort

	Hazard Ratio (95% CI) for melatonin		
**Outcome**	**No Lag time**	**Lag time: 1 year**	**Lag time: 2 years**
**Age-related cataract**	0.796 (0.741,0.855)	0.741 (0.681, 0.807)	0.826 (0.742,0.919)
**Other cataract**	0.555 (0.489,0.629)	0.503 (0.433, 0.584)	0.542 (0.448,0.655)
Traumatic cataract	0.376 (0.076,1.866)	0.840 (0.140, 5.045)	1.272 (0.179,9.058)
Complicated cataract	0.376 (0.076,1.864)	0.890 (0.148, 5.339)	0.414 (0.043,3.986)
Drug induced cataract	1.084 (0.153,7.711)	NA	NA
Secondary cataract	0.559 (0.440,0.710)	0.428 (0.321, 0.572)	0.477 (0.334,0.679)
Other specified cataract	0.663 (0.429,1.025)	0.804 (0.495, 1.309)	0.785 (0.430,1.435)
Unspecified cataract	0.532 (0.459,0.618)	0.498 (0.417, 0.594)	0.531 (0.425,0.663)

Table S4. Sensitivity Analysis of Cataract Risk in Melatonin Users Versus Non-Users

	Event number		
**Outcome**	**Melatonin cohort** (*n*= 34,951)	**Non-Melatonin cohort** (*n*= 34,951)	**Hazard Ratio** (95% CI)
**Age-related cataract**	1,449	2,208	0.785 (0.735,0.839)
**Other cataract**	1,397	1,911	0.885 (0.826,0.948)
Traumatic cataract	≤ 10	11	0.693 (0.268,1.790)
Complicated cataract	17	20	0.895 (0.468,1.710)
Drug induced cataract	≤ 10	≤ 10	3.227 (0.872,11.941)
Secondary cataract	832	1,123	0.842 (0.769,0.921)
Other specified cataract	154	198	0.851 (0.689,1.051)
Unspecified cataract	997	1,259	0.939 (0.864,1.020)

Figure S1. Schematic study design.

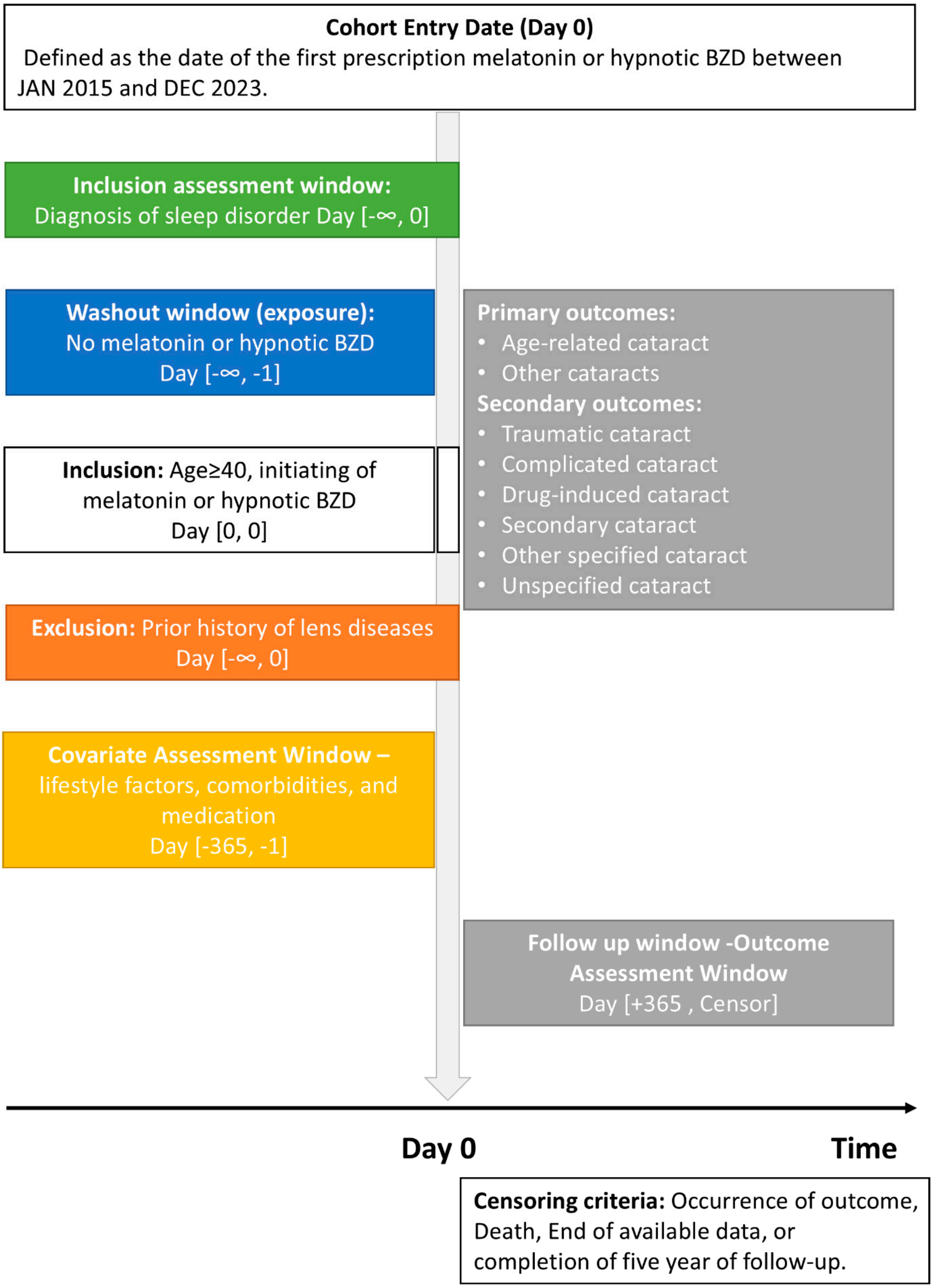

Supplement: Supplementary file 1 [file antioxidants-14-01016-s001.zip › antioxidants-3769330-supplementary.pdf]
